# Short-Term Fidelity, Habitat Use and Vertical Movement Behavior of the Black Rockfish *Sebastes schlegelii* as Determined by Acoustic Telemetry

**DOI:** 10.1371/journal.pone.0134381

**Published:** 2015-08-31

**Authors:** Yingqiu Zhang, Qiang Xu, Josep Alós, Hui Liu, Qinzeng Xu, Hongsheng Yang

**Affiliations:** 1 Key Laboratory of Marine Ecology and Environmental Sciences, Institute of Oceanology, Chinese Academy of Sciences, Qingdao, China; 2 University of Chinese Academy of Sciences, Beijing, China; 3 Department of Biology and Ecology of Fishes, Leibniz-Institute of Freshwater Ecology and Inland Fisheries, Berlin, Germany; North Carolina State University, UNITED STATES

## Abstract

The recent miniaturization of acoustic tracking devices has allowed fishery managers and scientists to collect spatial and temporal data for sustainable fishery management. The spatial and temporal dimensions of fish behavior (movement and/or vertical migrations) are particularly relevant for rockfishes (*Sebastes* spp.) because most rockfish species are long-lived and have high site fidelity, increasing their vulnerability to overexploitation. In this study, we describe the short-term (with a tracking period of up to 46 d) spatial behavior, as determined by acoustic tracking, of the black rockfish *Sebastes schlegelii*, a species subject to overexploitation in the Yellow Sea of China. The average residence index (the ratio of detected days to the total period from release to the last detection) in the study area was 0.92 ± 0.13, and most of the tagged fish were detected by only one region of the acoustic receiver array, suggesting relatively high site fidelity to the study area. Acoustic tracking also suggested that this species is more frequently detected during the day than at night in our study area. However, the diel detection periodicity (24 h) was only evident for certain periods of the tracking time, as revealed by a continuous wavelet transform. The habitat selection index of tagged *S*. *schlegelii* suggested that *S*. *schlegelii* preferred natural reefs, mixed sand/artificial reef bottoms and mixed bottoms of boulder, cobble, gravel and artificial reefs. The preference of this species for the artificial reefs that were recently deployed in the study area suggests that artificial seascapes may be effective management tools to attract individuals. The vertical movement of tagged *S*. *schlegelii* was mostly characterized by bottom dwelling behavior, and there was high individual variability in the vertical migration pattern. Our results have important implications for *S*. *schlegelii* catchability, the implementation of marine protected areas, and the identification of key species habitats, and our study provides novel information for future studies on the sustainability of this important marine resource in eastern China.

## Introduction

The historical exploitation of naturally reproducing fish populations has led to the overexploitation of many stocks and serious effects on natural systems, societies and economies worldwide [1–6]. A primary factor in the overexploitation of marine resources is poor spatial knowledge of fish stocks [7–11]. For example, fish movement is rarely considered in stock assessments, although movement is a key parameter in the population structure of many species (e.g., Atlantic bluefin tuna *Thunnus thynnus* [12]) and the potential survival benefits provided by marine protected areas (MPAs) [13]. An understanding of the spatial dimension of the fish stock is therefore integral to the sustainability of fisheries. Such understanding enables us to identify the expected distribution of resources or the consequences of selective fishing [14–16]. Unfortunately, proper fishery management is complex and requires involved spatial socio-ecological approaches to fully elucidate the role of space in fishery sustainability [17]. Because of its complexity, traditional fishery management is typically based on homogeneous scenarios, and the spatial component is frequently overlooked [7,18].

With the recent development of fish-tracking technology [19] and fishing vessel monitoring systems [20], ecologists and fishery scientists have gained new powerful tools to enhance the understanding of the spatial dimensions of fisheries. For example, an extensive study involving tracking *T*. *thynnus* with satellite tags provided a more detailed knowledge of the population structure to enable the management of naturally spawning stocks based on differences in fishing mortality between the Gulf of Mexico and the Mediterranean [12]. At a smaller spatial scale, acoustic tracking has emerged as a powerful tool for the estimation of spatial distributions and temporal patterns of coastal fish to inform proper spatial management of coastal fisheries worldwide [21]. Tagging and tracking coastal fish using an array of omnidirectional receivers provides detailed information on the movements of coastal fish [22] as well as their habitat utilization [10,23], site fidelity [24], vertical movement [25] and diel migration [26]. Elucidating these behavioral traits has aided the sustainable development of coastal fisheries, particularly by contributing to the delineation of MPAs and identifying essential habitat [13,21,27–29].

The spatial and temporal distribution of rockfish is especially relevant for the vulnerability of rockfish to overexploitation. Rockfish comprise a number of species within the genus *Sebastes* and are highly exploited in the Pacific Ocean [30]. The abundance of Puget Sound rockfishes has declined approximately 70% between 1970 and 2010 [31], and the bocaccio rockfish *Sebastes paucispinis* and the yelloweye rockfish *Sebastes ruberrimus* were at high and moderate risk of extinction throughout Puget Sound/Georgia Basin [32]. Rockfish are particularly vulnerable to overexploitation [33–35] for two main reasons. First, the life history of rockfish is characterized by long lifespans, late maturation, low offspring production (oviparous) rates and generally large adult sizes [33,36]. Second, rockfish have strong site fidelity and a limited capacity for movement, increasing their vulnerability to local depletion. In a 445-day study off the coast of central California, Green et al. [37] reported that the blue rockfish *Sebastes mystinus* only moved within a home area of 0.07 to 0.53 km^2^. Jorgensen et al. [38] concluded that *S*. *mystinus* has strong site fidelity and that short-term excursions were correlated with environmental factors. Hannah and Rankin [39] performed an extensive study of several rockfish species off the coast of Oregon, USA, and reported that five of the eight species examined were only detected by one or two nearby receivers in a large (5200-ha) receiver grid of 30 receivers during the entire year. These authors also provided substantial evidence for small vertical migrations [39], which have implication for rockfish conservation and its vulnerability to overfishing, as fish performing stronger vertical migrations are more prone to encounter fishing gears and be harvested [40]. Many studies [41,42] have therefore suggested that species of the genus *Sebastes* have strong site fidelity, which contributes to their vulnerability to local depletion and overexploitation [43].

Much less is known about the spatial and temporal dynamics of the movement behaviors of the black rockfish, *Sebastes schlegelii*. This species inhabits shallow coastal waters (less than 100 m deep) along the coasts of Japan, Korea, and northern China [44,45]. Rockfish catch, including that of *S*. *schlegelii*, declined from the 1980s to 2005 [45,46]. *S*. *schlegelii* is one of the most dominant fish species in the Yellow Sea (China) [35] and is estimated to be one of the most important targeted species at our study site in Haizhou Bay [47]. Because of intense bottom trawling, the catch per unit of effort (CPUE) and mean individual weight of *S*. *schlegelii* in the Yellow Sea have significantly declined since 1985 [35]. To recover overexploited stocks, China and Japan have implemented different management tools (such as prohibited fishing periods and zones and minimum sizes limitations) and a widespread stock-enhancement program for this species [44,48,49]. MPAs, such as the National Marine Protected Area of Demersal Fish Ecology in Lijin Dongying Shandong Province [50], have recently been created along the coast of the Yellow Sea to reduce fish stock mortality by prohibiting commercial and recreational fishing activities. In addition, the Shandong Provincial Ocean and Fishery Agency and the Rizhao Blue Economy Zone Agency instituted two scientific research programs in 2005 to enhance habitat recovery for *S*. *schlegelii* and the greenling *Hexagrammos otakii*, including the deployment of artificial reefs (ARs) around the Qiansan Islets [51–53]. The success of these two management measures (MPAs and ARs) is strongly dependent on the range of movement of this species [10,13], which is unknown.

In this study, we provide new information about the spatial and vertical behavior of *S*. *schlegelii*. We performed an acoustic tracking experiment offshore of Ping Island in Haizhou Bay, China, to provide new data on *i*) spatial movements and site fidelity in the acoustic array, *ii*) the pattern of habitat utilization on natural habitats and artificial habitats and *iii*) the vertical movement behavior of this species. Because of the current status of the *S*. *schlegelii* population in the Yellow Sea and the emerging interest of local authorities in spatial management, our work provides useful information for managers.

## Materials and Methods

### Ethics statement

*S*. *schlegelii* collection and tagging and the deployment of the receiver mooring system around Ping Island were permitted by the State Oceanic Administration People’s Republic of China and the Qiansan Islets Aquatic Products Development Co. Ltd. All procedures were performed according to the guidelines of the American Fisheries Society for the use of fishes in research [54]. The study was approved by the ethics committee of Institute of Oceanology, Chinese Academy of Sciences. Our study did not involve endangered or protected species, and no animals were sacrificed. Acoustic tags were attached to fish after anesthetization with MS-222, and all efforts were made to minimize fish handling and harm.

### 1. Study area

**Fig 1:**
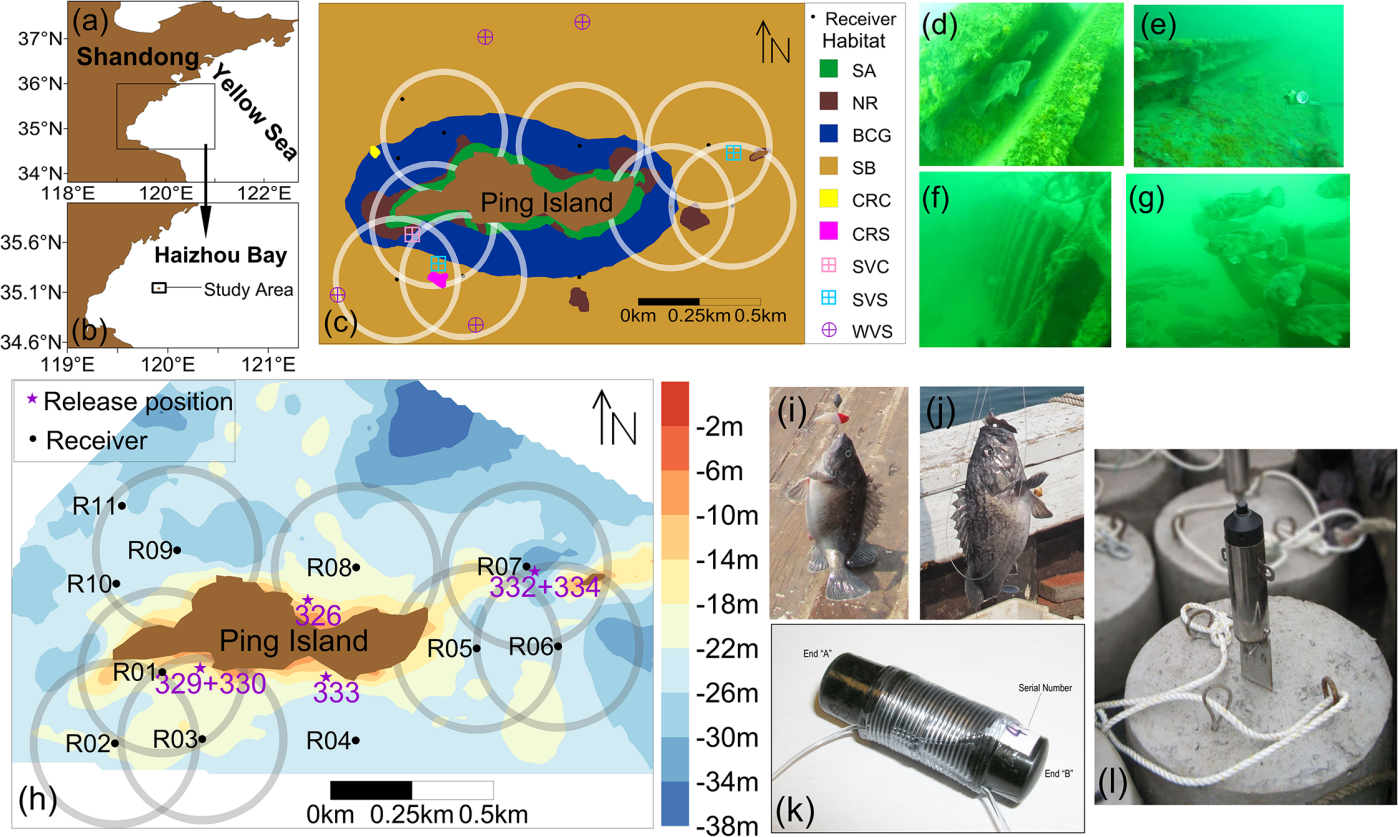
The study area location, habitat types, the tag wrapping method and the receiver mooring system. (a) The location of Haizhou Bay in the Yellow Sea. (b) The location of study area. (c) Bottom habitat types around Ping Island and detecting range of receivers. (d) Cement reef. (e)-(f) Sunken steel vessels. (g) Sunken wooden vessel. (h) The acoustic receiver array deployment, receiver positions and detecting range, the fish release positions and depth range (m) around Ping Island. (i)-(j) Two *S*. *schlegelii* captured via hook and line by local fishermen. (k) The wrapping method for attachment of the acoustic tags and (l) The omnidirectional receiver and the mooring system. In panel (c), the halos around receivers represent a 250-m detection radius, and 3 receivers (R04, R10 and R11) without halo were lost. Habitat are named as seagrass at rocky bottom dominated by *Sargassum sp*. (SA), natural reef (NR), boulder, cobble and gravel bottoms (BCG), sandy bottom (SB), cement reef at cobble bottom (CRC), cement reef at sandy bottom (CRS), sunken steel vessel at cobble bottom (SVC), sunken steel vessel at sandy bottom (SVS) and sunken wooden vessel at sandy bottom (WVS).

We performed an acoustic tracking experiment in the Qiansan Islets, a collection of three small islands in Haizhou Bay in the central Yellow Sea, Northwest Pacific Ocean (see Fig 1). Our study was centered around Ping Island (Fig 1), the largest islet of the Qiansan Islets. Our study area covered the depth from the water surface of Ping Island to 38 m, and included different types of *S*. *schlegelii* natural habitats: natural reefs and a mixture of kelp beds, gravel-cobble bottoms and sandy substrates (Fig 1). Local authorities have submerged large rocks, cement reefs, barge boats and fishing vessels to create artificial habitats to enhance marine stocks around the Qiansan Islets [51] (see Fig 1), and an MPA was created. The marine reserve surrounding Ping Island is the largest reserve within this MPA. We characterized the depth (m) of the study area using a multi-beam sounding system (see S1 Fig). The bottom substrate type was determined using an EdgeTech 4100 Side Scan Sonar System (EdgeTech, Massachusetts, USA) at 500 kHz (S2 Fig). The bottom types were further validated by the video surveys of the SeaBotix LBV150-4 (Teledyne SeaBotix, California, USA) and the dive operated stereo-video system (stereo-DOV) [47]. Video examples of the bottom types are provided in S1–S7 Videos. The spatial coverage of seagrass was quantified by a Simrad EY60 split-beam device run at 200 kHz along closely spaced routes parallel to the shelf in the shallow waters of the Qiansan Islets (< 20 m deep, see S3 Fig for visualization of the trajectory), and the results suggested that the seagrass was mainly distributed in shallower waters < 15 m deep (Fig 1 (C)). In general, the Qiansan Islets are dominated by a semi-diurnal tide, with a southwest-oriented flood current and a northeast-oriented ebb current at Ping Island (for more details, see [55,56]).

### 2. Receiver placement

On 1 August 2013, eleven acoustic receivers (model VR2W, VEMCO; Bedford, Nova Scotia, Canada) were deployed on the seabed around Ping Island (Fig 1) to record the horizontal and vertical movements of *S*. *schlegelii*. Due to the limited number of receivers and limited detection range in our study area (see Fig 1), the study area was divided into five regions: a southwest (SW) region that was monitored by three receivers (R01, R02 and R03), a southeast (SE) region that was monitored by one receiver (R04), an east (E) region that was monitored by three receivers (R05, R06 and R07), a northeast (NE) region that was monitored by one receiver (R08), and a northwest (NW) region that was monitored by three receivers (R09, R10 and R11).

To obtain the detection range of the VR2W receiver in our study area, an empirical range test was conducted using two anchored acoustic tags and one moving VR2W receiver. Two anchored acoustic tags (model: V9P-1H-A69-9002, 40 to 80 s burst interval) were fixed 10 m below the sea surface at the same position. The VR2W linked to the active VUE software (VEMCO) and situated 5 m below the sea surface was moved using a research vessel (equipped with an external GPS) from a distance of 0 m to 500 m from the two anchored tags. The received signal was at a maximum distance of 250 m from the receiver, which was calculated based on the GPS position where the receiver received signals and the acoustic tag position. Therefore, we considered that the maximum detection range was 250 m (Fig 1 panel (h)). This maximum range of detection was similar to that reported by Villegas-Ríos et al. [57] for the same tracking equipment in the shallow waters of Spain.

Before the acoustic receiver deployment in the study area, each VR2W receiver was embedded in a protective steel barrel that was fixed on a cement base to ensure stability (Fig 1). The fixed receiver system was placed on the sea floor using a heavy lift research vessel. Clements et al. [58], Simpfendorfer et al. [59] and Espinoza et al. [60] suggested that detection probability is highly influenced by environmental noise and may lead to erroneous interpretations of behavioral patterns. In fact, recent studies suggest that the detection of a distant acoustic signal can be highly influenced by environmental variables, such as the current generated by the tides [23,61,62]. For this reason, we studied the patterns of three control tags (V9-1H-A69-1601, 9 mm diameter, 42 mm length, weight in air 3.6 g, weight in water 2.2 g, power output 151 dB re 1 μPa at 1 m, 455 d life, 500 to 700 s burst interval) that were anchored at the centers of the SW array (ID: 5011), E array (ID: 5012) and NW array (ID: 5001) in the study area to accurately interpret the observed fish behaviors, following the recommendations of Payne et al. [63].

### 3. Tag attachment

Six *S*. *schlegelii* with weights ranging from 260 g to 610 g and total lengths ranging from 260 mm to 340 mm were sampled near the shelf of the study area without precise locations using conventional cage nets. These individuals were held for 24 h at the bottom of a fishing vessel in a recirculating live well (4 m × 2 m × 1 m). After 24 h, each individual was anesthetized using a dose of 100 mg/L MS-222. We then tagged the fish with an acoustic-pressure transmitter (model: V9P-1H-A69-9002, pressure sensitive [0 to 50 m] of 0.22 m resolution, 9 mm diameter, 42 mm length, weight in air 5.2 g, weight in water 2.7 g, power output 151 dB re 1 μPa at 1 m, 45 d life, 40 to 80 s burst interval) using an external attachment (Fig 1 (K)). This method of tagging is quick, easy to perform, and suitable for short-term studies [39,64]. A needle was used to thread the two ends of the cord through the membrane between the second and third dorsal fin spines and the membrane between the fourth and fifth spines. The cord ends were then threaded through two holes in a polyethylene gasket [65,66] on the opposite side of the fin and tied together. The total duration of the tagging process was approximately 2 min for each fish. After full recovery from anesthesia in the live well, the fish were released in the study area (see positions of release in Fig 1 (H)). After 60 days of receiver recording tagged fish underwater, we recovered the receivers, downloaded the data and constructed a geo-referenced database that included the data from tagged fish, the receiver positions and the time-related acoustic detections.

### 4. Data analysis

We structured the data analysis to achieve the three major objectives of this study: *i*) the site-fidelity and temporal behavior variability of *S*. *schlegelii*; *ii*) the spatial patterns and habitat utilization of *S*. *schlegelii*; and *iii*) the water column use and vertical behaviors of *S*. *schlegelii*. Detection data were filtered to remove potential spurious detections, defined as any single transmitter code occurring alone during a 24-h period [57,67]. Given that the fine-scale movement could not be determined, the geo-referenced receiver location was used as fish position, and the detecting range of the receiver was interpreted as the activity zone of the fish when the receiver picked up a tagged fish [68]. This approach is widely used when the number of receivers [10,69] or the expected horizontal shift is limited (e.g., other *Sebastes* species [39]). We derived a residence index for each individual following Green et al. [41] and March et al. [70] by calculating the total number of days a tagged fish was detected in a region of the receiver array (DD) firstly and then dividing DD by the total period (TP) from release to the last detection of each individual.

The effect of temporal variations on *S*. *schlegelii* behavior was assessed via a continuous wavelet transform (CWT) to identify any periodicity pattern in the hourly detections at different time scales (2, 4, 6, 16, 24, 32 and 64 hours). The robust predictive ability of CWT makes it one of the most promising tools for depicting multi-scale animal behaviors that are related to environmental properties, i.e., diel and tidal patterns [71]. CWT allows a time series to be divided into time-frequency space, which provides an alternative to Fourier analysis or other time-frequency decomposition methods [72] to identify temporal patterns in behavioral data during a tracking period [70,73]. The library *sowas* in R (http://www.r-project.org/) was used to compute the two-dimensional wavelet spectrum and to perform a point-wise test at a 95% significance level for each of the tagged fish and control tags [74]. We fitted a single CWT for each tagged individual and also for every control tag to separate any environmental periodicity from behavioral periodicities [63].

We also specifically tested how diel phase (day versus night) affected hourly detections of control tags and tagged fish. Hourly detection data were grouped based on tag ID, diel phase (day versus night) and tracking date. Diel phase was considered as an independent variable and treated as a fixed factor, while controlling individual differences by treating tag ID as a random factor. For this assessment, we fitted the generalized linear mixed model (GLMM) to the data of hourly detections for control tags and tagged fish using the *lme4* package in R [75]. The temporal autocorrelation of the GLMMs residuals was assessed using the autocorrelation function acf in the *stats* package in R. The GLMMs were computed using the Poisson distribution, and the dispersions of the GLMMs were computed to test the distribution fitness (Poisson distribution requires dispersion to be 1 [76]). The GLMM of control tag detections was fitted well by a Poisson distribution (dispersion = 0.98), no significant temporal autocorrelation occurred, and the parameters of the GLMM were calculated. However, the GLMM of tagged fish hourly detections suffered strong over-dispersion (dispersion = 10.85) suggesting a zero-inflation of the data. We then fitted three zero-inflated GLMM with Gaussian, Poisson and negative binomial distributions using the package ‘glmmADMB’ in R. The Akaike information criterion (AIC) of the three zero-inflated GLMMs was calculated using the *bbmle* package in R. The zero-inflated negative binomial GLMM model was of the lowest AIC and fit best [77]. Temporal autocorrelation was detected in the residuals of the model. The data were then resampled randomly until the acf plot showed no significant temporal autocorrelation. The parameters of the zero-inflated negative binomial GLMM with no significant autocorrelation were used to test whether diel phase affected hourly detections of tagged fish.

*S*. *schlegelii* spatial behavior was assessed by examining temporal plots of detections and residence time at specific receivers. We also determined *S*. *schlegelii* preference for certain habitats by calculating a habitat selection index (HSI) value for each fish to quantify preference for/avoidance of different habitat types [78]. For each fish, the HSI value was calculated as the quotient of habitat utilization, defined as the percentage of detections in a specific habitat, and habitat availability, defined as the percentage of each habitat type in the 250 m detecting range of the receivers that detected the fish [78]. Thus, preference or avoidance was calculated as the degree of deviation (in a positive or negative direction, respectively) from the value of 1 [73,78].

We used the Tide_pred.m script (function) in the Tide Model Driver (TMD) MATLAB package [79] to predict the tide height at Ping Island during our tracking period. Depth values collected from tagged fish were closely related to predicted tide height (S4 Fig and S1 Table). CWT for fish depths revealed significant semi-diurnal periodicity (12 h) (S5 Fig), similar to the local semi-diurnal tide periodicity. To ascertain accurate depth and vertical movements of *S*. *schlegelii*, the fish depths were corrected by deducting the predicted tide height (m) from the collected depth every minute. The effect of temporal variation on the vertical behavior of *S*. *schlegelii* was statistically assessed via CWT to detect any periodicity in depth at different time scales (2, 4, 6, 16, 24, 32 and 64 hours). Daily vertical movements of *S*. *schlegelii* were also grouped into one of 3 mutually exclusive categories: large vertical movement (LV), small vertical movement (SV) and bottom dwelling behavior (BD), as described by Parker et al. [80]. LV was characterized as >5 m of vertical movement per day. SV consisted of vertical movements between 1 and 5 m per day. BD was characterized as <1 m vertical movement per day at a steady depth or the depth of a semi-diurnal tide pattern.

## Results

### 1. Detection sample size and site fidelity

**Fig 2:**
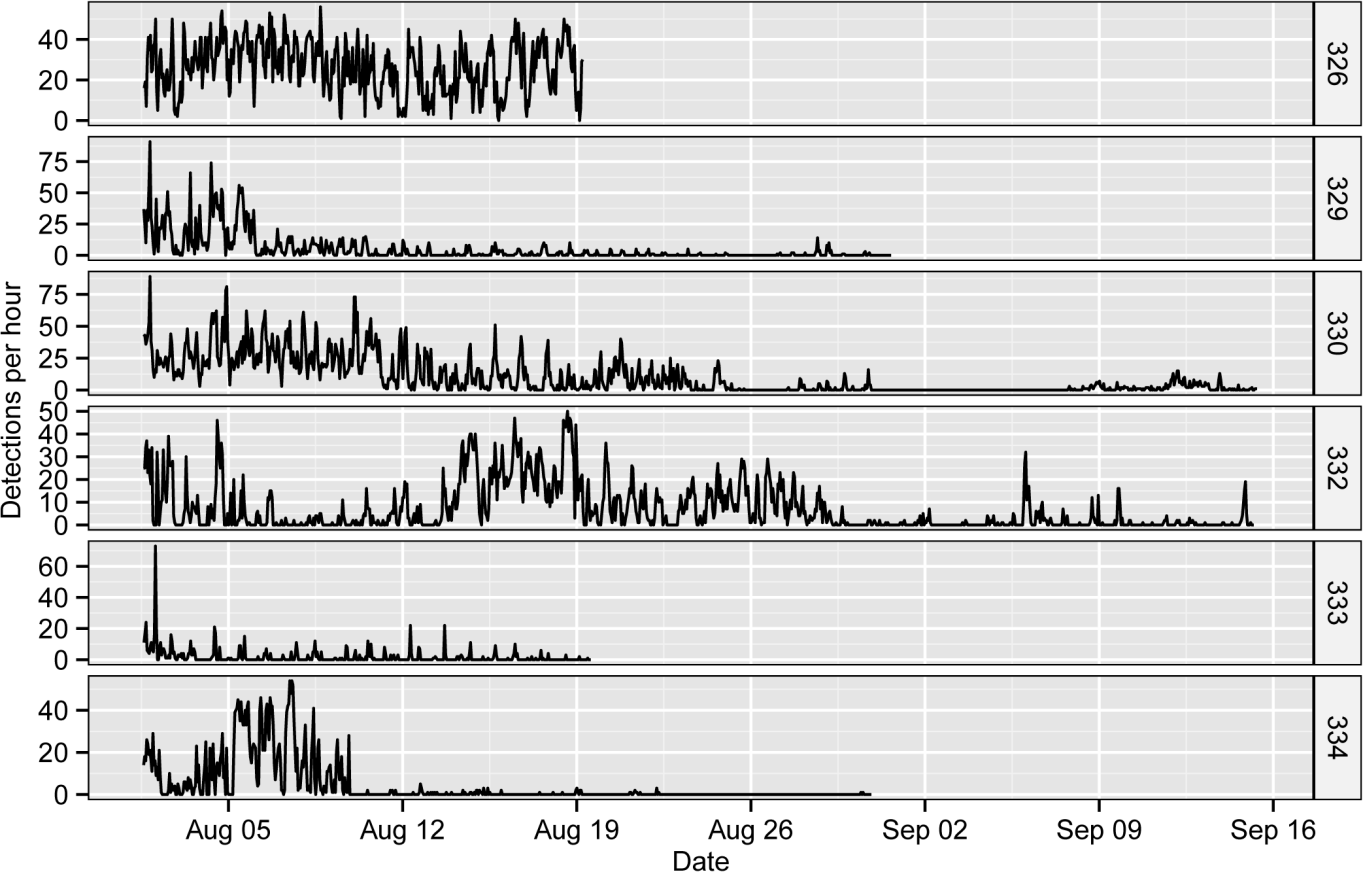
Chronograms of the hourly detections (pooled from all receivers) of the six tagged *S*. *schlegelii*.

**Fig 3:**
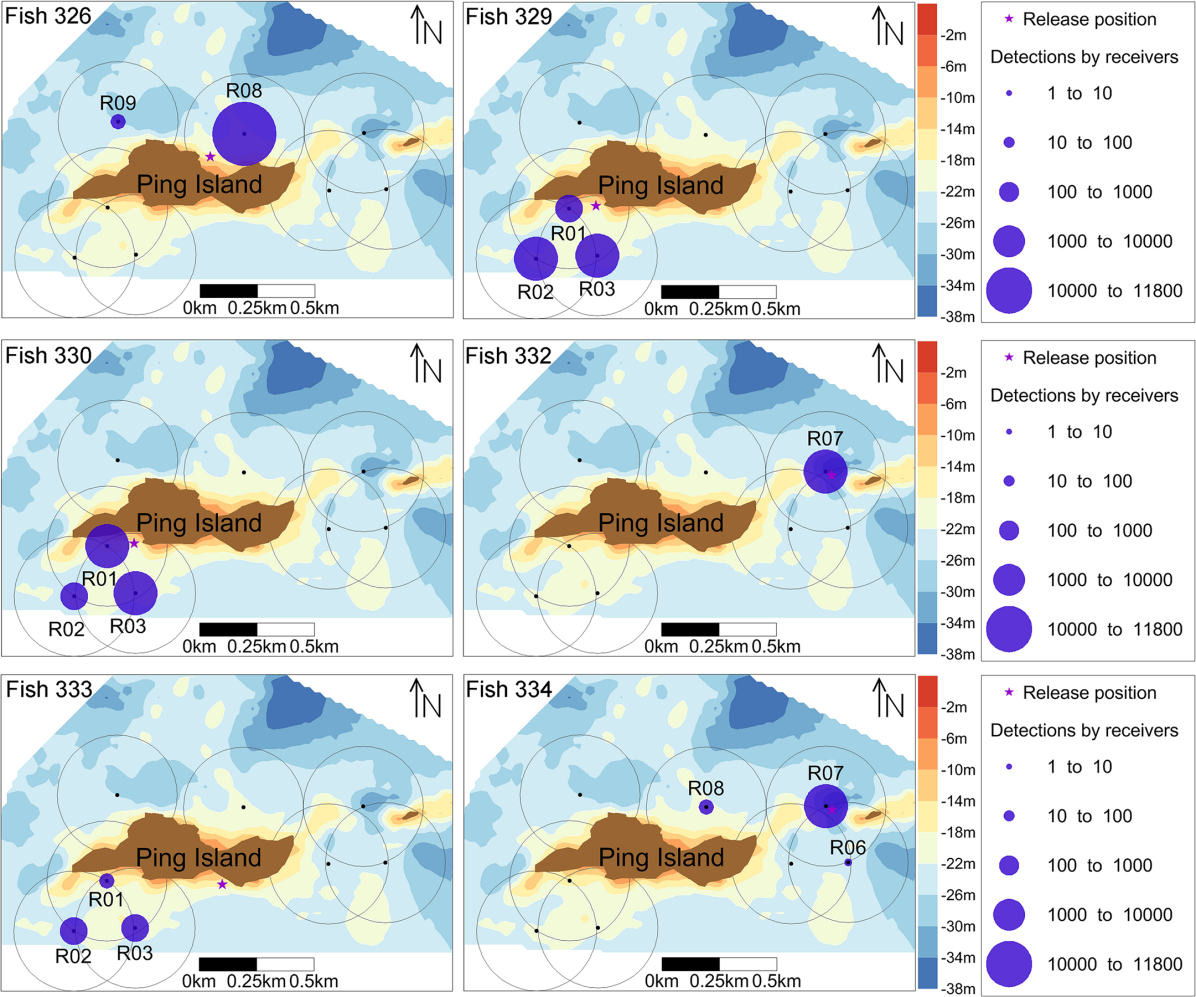
Spatial distribution of six tagged *S*. *schlegelii* detections at receivers. The size of blue circles represents the number of individual detections of six tagged *S*. *schlegelii* by every receiver.

A total of 36,389 acoustic detections were collected from the six tagged fish in our acoustic tracking experiment at Ping Island (Fig 2). The mean (± s.d.) number of detections of each fish was 6065 ± 4241 and ranged from a minimum of 791 (ID: 333) to a maximum of 11201 (ID: 326). The fish were detected by at least one (ID: 332) and a maximum of 3 receivers (ID: 329, 330, 333 and 334), and fish 326 was detected by two receivers (Fig 3). The total DD of the tagged fish ranged from 19 to 46 days, with an average of 28.67 ± 10.97 days. One fish (ID: 332) was detected in varying hourly detections throughout the entire study period (1 August to 15 September), a total of 46 days (Fig 2). Another fish (ID: 330) was detected for 38 days but was lost from 30 Aug to 7 Sep, with a total period of 46 days. Fishes 329 and 334 were detected before 31 Aug for 30 days and 21 days, with total periods of 31 days and 30 days respectively. Fishes 326 and 333 were detected continuously until 19 August, a total of 19 days. The residence index of tagged fish in the whole receiver array ranged from 0.70 to 1.00, with an average of 0.92 ± 0.13 (Table 1). Four of six tagged fish were detected in only one region of the study area and exhibited different degrees of site fidelity in their particular region (Table 1). Fish 326 was mainly detected in the NE region (R08), fish 329, 330 and 333 were only detected in the SW region (R01, R02 and R03), and fish 332 was only detected in the E region (R07), the deepest position of the acoustic array (Fig 1 (H)).

**Table 1:** Biological information and residence indices of six tagged *S*. *schlegelii* individuals.

ID	TL	W	RP	TDD	TP	TRI	SW region		E region		NE region		NW region	
	mm	g		d	d		DD	RI	DD	RI	DD	RI	DD	RI
**326**	340	550	NE	19	19	1.00					19	1.00	8	0.42
**329**	285	400	SW	30	31	0.97	30	0.97						
**330**	260	320	SW	38	46	0.83	38	0.83						
**332**	260	330	E	46	46	1.00			46	1.00				
**333**	320	610	SE	19	19	1.00	19	1.00						
**334**	255	260	E	21	30	0.70			9	0.30	12	0.40		

TL, total length; W, weight; RP, release position; TDD, total number of days detected by all receivers; TP, the total period from release to the last detection; TRI, total residence index; DD, number of days detected by an array; RI, residence index in an array.

### 2. Diel detection pattern

**Fig 4:**
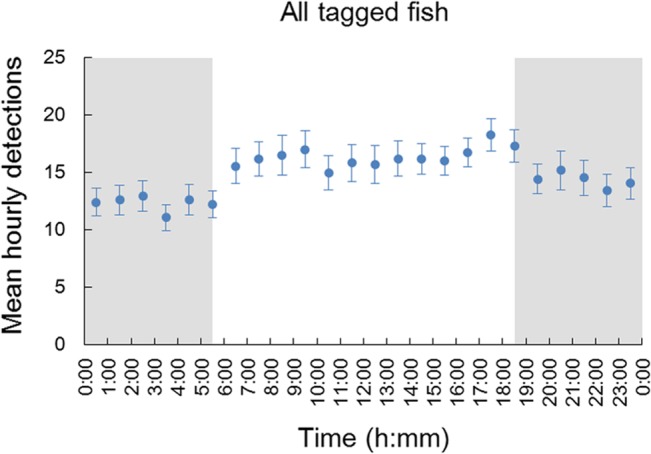
Mean hourly detections (± SE) of six tagged fish. Background color indicates day (white) or night (grey) based on the local sunrise and sunset time.

The average hourly binned detections plot revealed a daily pattern of a higher number of detections during the day (15.54 ± 14.768 h^−1^) than during the night (13.07 ± 13.562 h^−1^) (Fig 4). The GLMM (zero-inflated negative binomial model) confirmed this pattern and indicated that the hourly *S*. *schlegelii* detections varied significantly with diel phase (day versus night) (*P* < 0.01) (Table 2). The GLMM fitted to the detections of control tags did not show changes between day and night (Table 2), which excluded any environmental cause for the results observed in the tagged fish.

**Table 2:** Results of generalized linear mixed model testing effect of diel phase (day vs. night) on the hourly detections of control tags (in Poisson distribution) and tagged *S*. *schlegelii* (in zero-inflated negative binomial distribution).

	Estimate	Std. Error	z value	*P* (> |z|)
Control tags				
(Intercept)	0.53	0.17	3.04	<0.01
day vs. night	0.07	0.06	1.16	0.247
Tagged fish				
(Intercept)	2.59	0.18	14.1	<0.001
day vs. night	-0.13	0.05	-2.6	<0.01

**Fig 5:**
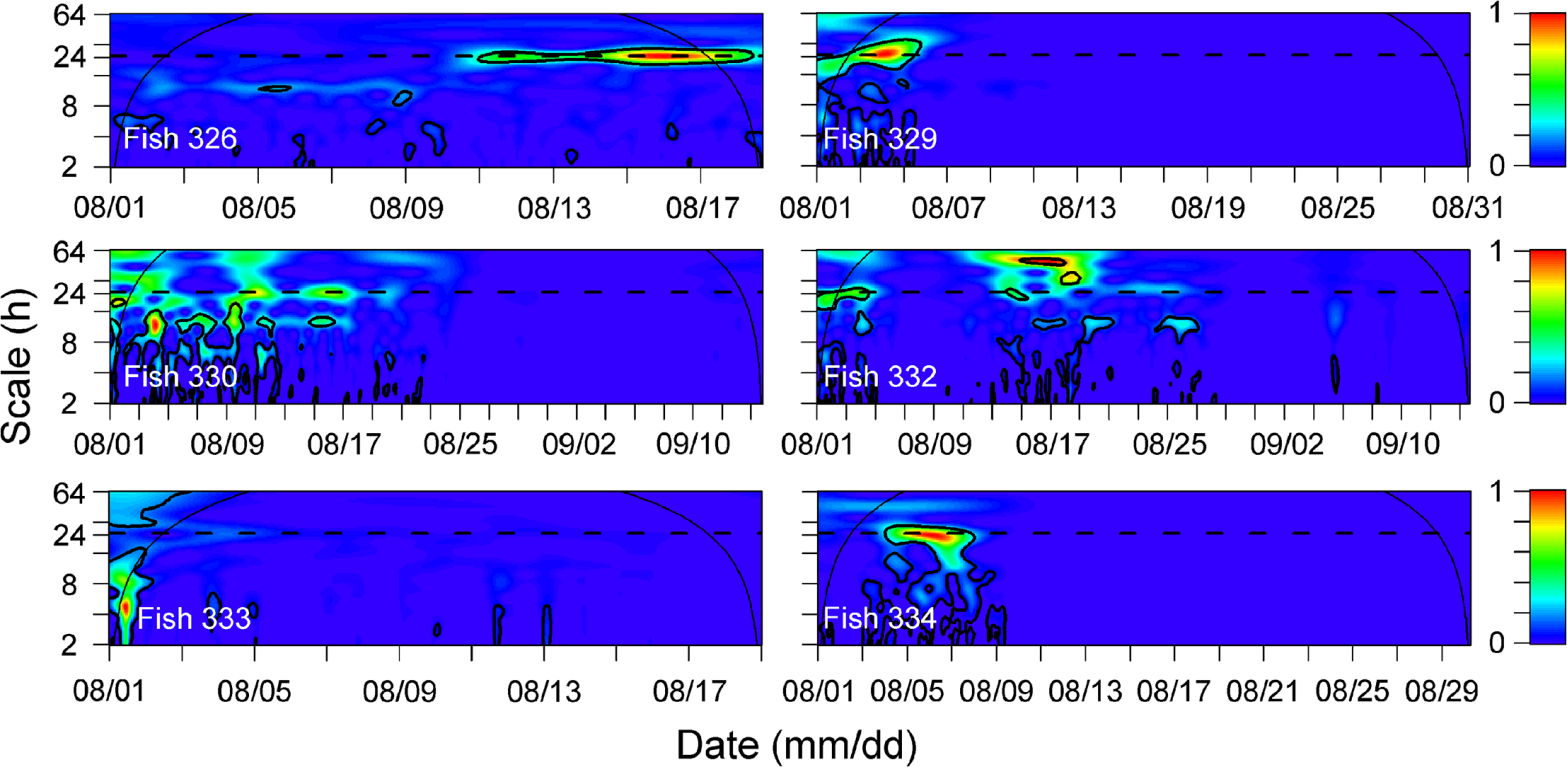
Wavelet sample spectrums using a Morlet wavelet for detections of 6 tagged fish. Periodicities were detected in 3 individuals on a 24 h scale (horizontal dashed line). Continuous thin lines represent the cone of influence (COI). Values outside the COI should not be interpreted due to edge effects. Thick contours represent the 95% confidence level and scale bar represents the intensity of the time-frequency space over time.

**Fig 6:**
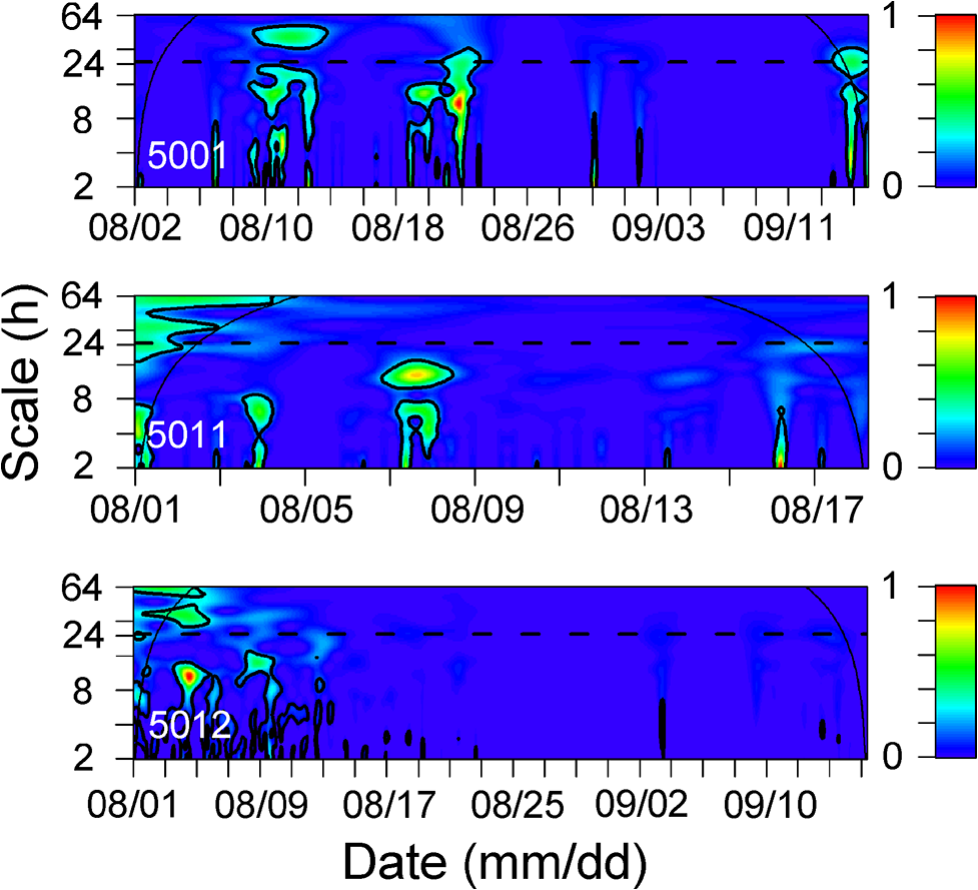
Wavelet sample spectrums using a Morlet wavelet for 3 control tags. Continuous thin lines represent the cone of influence (COI). Values outside the COI should not be interpreted due to edge effects. Thick contours represent the 95% confidence level and scale bar represents the intensity of the time-frequency space over time. The pattern without diel periodicity is indicated by the lack of thick contours on a 24 h scale (horizontal dashed line).

The temporal pattern of detections was assessed for every fish using wavelet spectrums (Fig 5). The diel pattern observed was only evident for certain periods of time and was not consistent across all tracking periods. The pattern of diel periodicity (24 h) was indicated by significant patches at 24 h scale in wavelet spectrums (Fig 5). For example, diel (24 h) periodicity was only evident after 11 August for fish 326, between 4 and 9 August for fish 334 and after tagging for fish 329 (Fig 5). The CWT results for the control tags confirmed that the pattern of significant diel periodicities was related to fish behavior but not environmental factors (see the absence of significant patches at 24 h scale in Fig 6).

### 3. Habitat use

Most acoustic detections of individual fish were recorded by a relatively small number of receivers, suggesting high site fidelity to specific areas (Fig 3). Habitat availability, defined as the percentage of each habitat type in the range of the receivers that detected the fish, varied among habitat types (presented in Table 3). The habitat type of the greatest availability for tagged *S*. *schlegelii* at our study site was mixed sand/artificial reef bottoms (SB + AR) (69.17 ± 12.67%), followed by sandy bottom (SB) (29.24 ± 22.91%), mixed bottoms of boulder, cobble, gravel and artificial reef (BCG + AR) (27.36 ± 0%), boulder, cobble and gravel bottoms (BCG) (19.43 ± 17.91%), seagrass (dominated by *Sargassum* sp.) (SA) (5.55 ± 2.26%) and natural reef (NR) (4.14 ± 1.06%). The average (± s.d.) habitat selection index (HSI) was larger than 1 (i.e., preference) for NR (1.11 ± 0.42), BCG + AR (1.05 ± 0.38) and SB + AR (1.04 ± 0.25) but less than 1 for SA (0.98 ± 0.71), BCG (0.79 ± 0.29) and SB (0.53 ± 0.62). These results suggest that fish exhibit a preference for NR, BCG + AR and SB + AR habitats and avoid SA, BCG and SB habitats. The HSI of habitats that included AR (i.e., BCG + AR and SB + AR) was always higher than 1, suggesting that *S*. *schlegelii* may prefer these artificial habitats.

**Table 3:** Habitat selection index and available habitat area percentage for each tagged individual.

ID	Habitat selection index (available habitat area (%))					
	SA	NR	BCG	BCG + AR	SB	SB + AR
**326**	1.19 (9.36)	1.50 (5.62)	0.92 (39.59)		0.97 (45.44)	
**329**	0.93 (5.03)	0.90 (4.12)		1.00 (27.36)		1.01 (63.48)
**330**	2.02 (5.03)	1.69 (4.12)		1.46 (27.36)		0.68 (63.48)
**332**		1.00 (2.33)	1.00 (5.37)			1.00 (92.30)
**333**	0.65 (5.03)	0.99 (4.12)		0.70 (27.36)		1.16 (63.48)
**334**	0.09 (3.28)	0.55 (4.54)	0.46 (13.34)		0.09 (13.04)	1.37 (65.80)

SA, seagrass (dominated by *Sargassum* sp.) at rocky bottoms; NR, natural reef; BCG, boulder, cobble and gravel bottoms; BCG + AR, mixed bottoms of boulder, cobble, gravel and artificial reef; SB, sandy bottom, SB + AR, mixed sand/artificial reef bottoms.

### 4. Vertical movement

**Fig 7:**
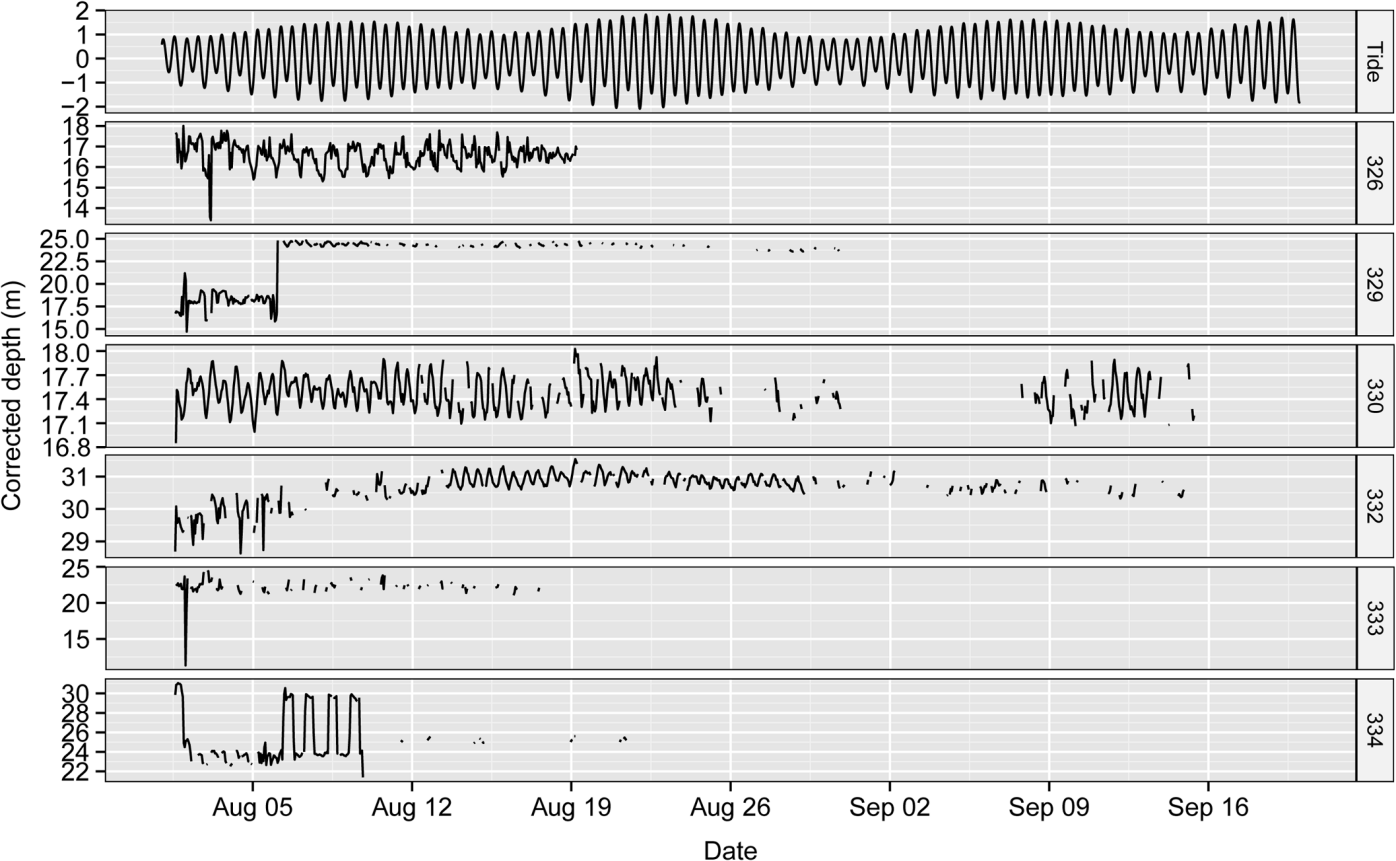
Chronogram of tide height and corrected depth of tagged *S*. *schlegelii* over the tracking period.

**Fig 8:**
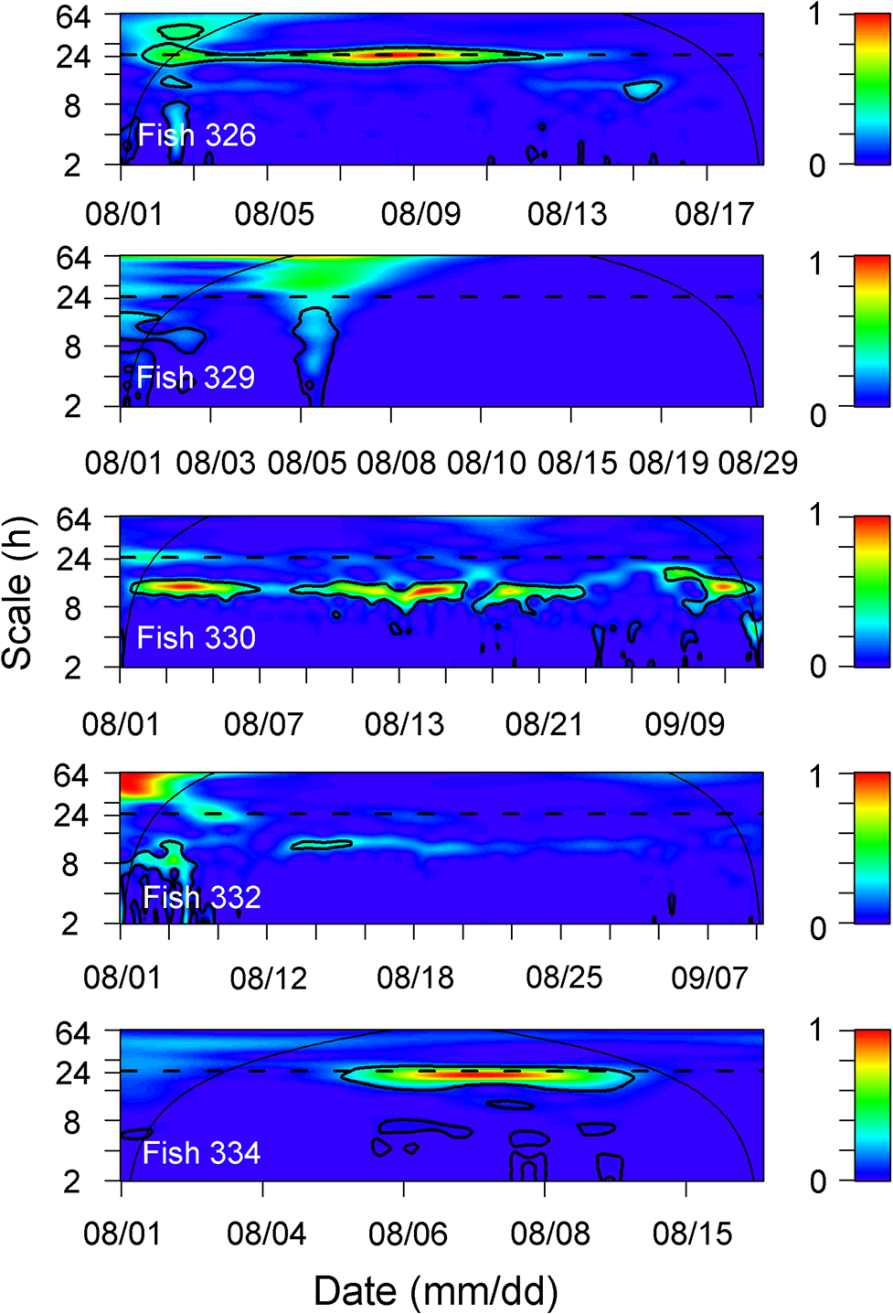
Wavelet sample spectrums using a Morlet wavelet for corrected depths of 5 tagged individuals.

Although the available depth of the study area ranged (within the detection range) from land (0 m) to 36 m, the tagged *S*. *schlegelii* individuals were detected at an average corrected depth of 20.90 ± 5.71 m (Fig 7). The shallowest fish (ID 326) was detected at the depth (16.64 ± 0.64 m), and the deepest fish (ID 332) was detected at the depth (30.72 ± 0.51 m). The temporal pattern of corrected depth (i.e., depth after correction for the height of the tide) was determined for each tagged fish using CWT and showed a high variability among individuals and a lack of any general pattern (Fig 8). CWT revealed significant diel periodicities (24 h) for fish 326 and 334, and semi-diurnal periodicities (12 h) for fish 330 (Fig 8). CWT showed no periodicity in vertical movement of fish 329 and 332.

Of the three vertical behavioral patterns (LV, SV and BD), BD was observed most often (Table 4). BD was the most common behavior for most tagged fish. Fish 329, 330 and 332 performed BD and SV for most of the tracking period. Between 1 August and 19 August, SV was the most common behavioral pattern of fish 326 and 333. In our tagged fish (ID: 326, 329 and 334), LV behavior was characterized by an ascent to shallow water during the night followed by a descent to deep water during the day. Fish 326 tended to ascend to shallow water and descend to deep water during tidal movements throughout the study (Fig 7). Fish 334 remained at a constant depth during the night but descended to the same maximum depth during the day for less than one week (5 Aug to 10 Aug) (Fig 7). Similarly, fish 329 tended to ascend to shallow water at night (1 Aug to 6 Aug).

**Table 4:** Total number of days that tagged *S*. *schlegelii* exhibited each type of vertical movement behavior.

ID	LV	SV	BD
326	4	15	0
329	3	3	24
330	0	3	35
332	1	9	36
333	1	17	1
334	5	4	13

LV, large vertical movement behavior; SV, small vertical movement; BD, bottom dwelling behavior.

## Discussion

In this study, we used acoustic tracking to provide new information about the spatial and temporal behaviors of *S*. *schlegelii*. We provide new support for site fidelity, habitat utilization and diel behavioral patterns over a short time scale (up to 46 days). In a recent effort to enhance stocks and reduce fishing mortality of *S*. *schlegelii*, the Japanese and Chinese governments have stipulated spatial management plans, including the delimitation of MPAs and the deployment of artificial reefs [44,48,49]. The success of these two management tools is strongly linked to the spatial and temporal patterns of exploited species. Therefore, our results provide information that can be directly applied to management plans for *S*. *schlegelii* and contribute to sustainable fishery development.

### 1. Site fidelity

Species of the *Sebastes* genus exhibit strong site fidelity and very small home ranges in coastal areas [38,39,81]. Our results indicate that *S*. *schlegelii* also exhibits strong site fidelity, with residence indices ranging from 0.70 to 1.00. The average residence index of tagged fish in our study area was 0.92 ± 0.13, and most of the tagged fish (with the exception of fish 333) were continuously detected for more than 19 days in the receiver array during 46 day detecting periods. Two tagged fish (ID: 330 and 332) were detected for nearly the entire tracking period and four tagged fish (ID: 326, 329, 333 and 334) were lost during the tracking period. One tagged fish (ID 334) migrated an approximate distance of 550 m from its site of release to a different receiver. The observation of distance migrated is similar to those reported for *S*. *schlegelii* [82] and *S*. *mystinus* [37]. The high site fidelity of *S*. *schlegelii* suggests that the MPAs recently created by the Chinese and Japanese governments may be effective in protecting these species from fishing mortality [45,51].

Variations of site fidelity and total detecting period among individuals may be attributed to fish behavior or to the limitations of the number of receivers in this study. *Sebastes* species exhibit a strong affinity for heterogeneous and structured rocky habitat [38, 39], and another black rockfish, *Sebastes inermis*, shelters in rock crevices during the day [42]. The use of structured habitat may result in a decline in detections. If the use of structured habitat was prolonged (e.g., if individuals remained in the rock crevices all day), this would have led to an observed but false reduction in the site fidelity index. The use of structured habitat may therefore explain why some tagged fish, such as fish 332, were detected for the entire tracking period but had an unexplained dramatic drop in detection during some periods (e.g., 5/08, 12/08). An alternative behavioral explanation for the site fidelity variation and the disappearance of tagged fish from the acoustic array is that *S*. *schlegelii* may use spatial areas larger than those covered by our receiver array. Although highly infrequent, we observed a maximum horizontal distance of 550 m in our study. In fact, Kang and Shin [82] reported that *S*. *schlegelii* moved 2 km away from their release points to regions between small islands or coastal areas. Being fished out may be another reason for the perpetual disappearance during the tracking period. The site fidelity and spatial utilization of the species should be assessed further with more sophisticated tracking experiments, including greater coverage of the tracking area and an increased number of tagged fish, on which our work provides a good guideline.

### 2. Diel detection pattern

A common feature of the spatial and temporal behavior of rockfishes of the genus *Sebastes* is that their behaviors differ between day and night [83,84]. This divergent behavioral pattern suggests that rockfish activity is closely related to ambient light intensity and that sunset and sunrise determine changes in behavioral state [38]. However, this pattern is not consistent among *Sebastes* species; some species are more active during the day, others during the night, and some exhibit no diel behavioral patterns. For example, the rosethorn rockfish *Sebastes helvomaculatus* exhibits a clear diurnal activity pattern (active during the day and inactive at night) [85]. Similarly, *S*. *mystinus* is more active during the day than at night, as indicated by a steep decrease in detection rates at dusk and resuming to the high level at dawn [38]. By contrast, *S*. *inermis* remains buried in rock crevices during the day but ranges between the surface and very deep depths at night [42]. In the present study, *S*. *schlegelii* was more frequently detected during the day than night, which is interpreted as a day active behavior [70].

However, the diel detection pattern of *S*. *schlegelii* was neither as evident nor as strong as those observed for *Sebastes* spp. or other species (e.g., *S*. *mystimus* [38] and *Serranus cabrilla* [73]). The CWT of tagged individuals indicates that the diel pattern was not consistent throughout the study period and suggests that the diel pattern is weak in this species. Different reasons could explain this result. First, this inconsistency may be due to the high relief AR habitat at our study site. The diel pattern of *Diplodus sargus* differs from a diurnal pattern (higher detections in daytime than during the night) at natural reefs to a nocturnal pattern (higher detections in the night than during the day time) at ARs [10]. These authors further inferred that NR resident fish search for food during the day and shelter in crevices during the night, while the AR resident fish were active during the night and rested in the day [10]. Second, the variation in diel pattern detection may be caused by diel vertical movement. For a period of one week, LV was observed for fish 326, 329 and 334 in our study after correcting depth by tide height (Fig 7 and Table 4). *S*. *schlegelii* moved to shallow water at night but returned to the deep water (i.e., closer to the receivers) during the day, which may have contributed to the higher hourly detections during the day compared to night. Unfortunately, with our experimental approach, we can not provide robust cause-and-effect evidence for this observed diel behavior, and we need further fine-scale tracking information to provide the exact mechanism generating these day and night differential behaviors that our approach has revealed.

### 3. Habitat use

We identified habitat preferences/avoidances for those periods during which fish were detected using HSI, under the limitation that the receiver detecting range was interpreted as the fish activity zone. HSI has been successfully used to describe the habitat preferences of various species. For example, Topping et al. [78] observed that California sheephead *Semicossyphus pulcher* favor habitats covered by boulders and rock walls rather than mud or sand. Alós et al. [73] established that *S*. *cabrilla* spends much more time on soft, gravel and detritus bottoms than in seagrass meadows (i.e., *Posidonia oceanica* beds). In this study, we provide some new information about the habitat utilization of *S*. *schlegelii*, on those habitats that include ARs. NR habitats and those habitats including ARs (i.e., BCG + AR and SB + AR) produced higher positive HSI values, suggesting that the preference of *S*. *schlegelii* for ARs is second only to that for natural reefs.

Habitat enhancement via the deployment of ARs has been a widely used management and conservation tool worldwide [86,87]. There is strong evidence that the implementation of ARs increases the local abundance of fishes by various mechanisms, creating a mating/spawning area, increasing fish growth and survival of juveniles, and attracting fish from outside the area [88–90]. AR restoration has been shown to mitigate rockfish population loss in many studies [91–93] and, in general, rockfish habitat utilization is closely linked to habitat complexity [81]. In fact, rockfish exhibit a strong affinity for rugged geomorphological features [38,94,95] and the heterogeneous and structured habitat of rocky substrates [39,96,97]. Reynolds et al. [81] demonstrated the potential of ARs to provide quality habitat for *S*. *caurinus* along the Alaskan convergent transform plate boundary. The results of our study agree with these previous findings for other species, and we suggest that ARs create quality habitat and serve as attractants for *S*. *schlegelii*.

### 4. Vertical movement

The results on the water column use indicate that *S*. *schlegelii* are mainly found at an average depth of 20.90 m (ranging from 16 m to 31 m). *S*. *schlegelii* is a demersal rockfish and remained closer to the bottom for a large portion of the tracking period in our study (Table 4). In another study, the black rockfish *Sebastes melanops* also remained close to the bottom; the proposed explanation for this behavior was that it enabled fish to avoid the water turbulence from wave action and storm activity [41].

Vertical movement of *S*. *schlegelii* (corrected by tide height) was highly variable among diel pattern (24 h periodicity), semi-diurnal pattern (12 h periodicity) and no periodicity pattern, as revealed by CWT. The observed patterns were separate from any other environmental effects, as the CWT of control tags did not exhibit any periodicity in the probability of detection. The diel pattern of vertical movement has been observed for many different species of *Sebastes* genus. For example, *S*. *paucispinis* and *S*. *mystinus* exhibit frequent vertical movements in shallow water during the day but move to deeper water and remain sedentary during the night [98,37]. *S*. *inermis* are in the shallow water around rock crevices during daytime and make frequent vertical movements at night [42]. Diel vertical migrations of *S*. *melanops* occurred for more than a week, with individuals being shallower either during the day in spring or at night in autumn [80]. Without considering the area lag in predicted tide height of our small study area, the semi-diurnal pattern of corrected depth may be caused by the location-dependent component of tide height.

For the three periodical patterns of *S*. *schlegelii* vertical movement, we can not fully identify the exact mechanisms. In fact, considering the limitations of our experimental design, the vertical behavior observed in *S*. *schlegelii* can be caused by vertical and horizontal displacement as observed in *S*. *inermis* [42]. But our study has revealed that the population of *S*. *schlegelii* could show a high degree of individual variability in vertical behavior. The individual pattern of vertical migration is an important component defining the vulnerability of an individual fish to the fishing gear. For example, Olsen et al. [40] found that Atlantic cod *Gadus morhua* individuals performing stronger diel migrations were more prone to be captured than those individuals with less evident migrations. The vertical movement of *S*. *schlegelii* should be studied further with increased tagged fish and fine scale movement to test whether the individuals with stronger vertical migrations are more susceptible to be captured as in the case of *G*. *morhua*, and whether vertical migration affects the population dynamics due to fishing activity.

Finally, our work has provided some new information on temporal and spatial behaviors of *S*. *schlegelii*, which is heavily exploited in Japan and China. Some additional topics about *S*. *schlegelii* movement behavior are expected to be studied further to promote the sustainability of the fishery: 1) the effective size of an MPA for *S*. *schlegelii*; 2) the diel activity levels of *S*. *schlegelii* at heterogeneous bottoms (e.g. AR); 3) the mechanisms responsible for vertical movements of *S*. *schlegelii* and the contribution of vertical migrations to overexploitation vulnerability for this species. An effective and successful MPA should be maximized and cover the area that is primarily used by the exploited fish [13], and therefore there is a need for a long-term tracking experiment using a large number of receivers and fish tags to determine the space utilization of this species over the large part of its lifespan. Fish are expected to vary in behavioral types [99], and further examination of the consistency and repeatability of these diel behaviors and vertical movements would establish whether there are different *S*. *schlegelii* behavioral types and the cause of those behaviors, which have strong implications for fisheries sustainability [100]. This study is a useful baseline for ecologists and fishery scientists in future acoustic tracking experiments that will address these questions. These results greatly contribute to our understanding of this economically important and vulnerable species.

## Supporting Information

S1 Fig The depth around Ping Island surveyed by multi-beam sounding system. (TIF)

S2 Fig The bottom types determined by EdgeTech 4100 Side Scan Sonar System. (TIF)

S3 Fig The dense routes of seagrass survey using Simrad EY60 split-beam at 200 kHz. (TIF)

S4 Fig Chronogram of depth collected from fish tags over the tracking period. (TIF)

S5 Fig Wavelet sample spectrums using a Morlet wavelet for depths of 5 tagged individuals. (TIF)

S1 Table The linear mixed model (in the *lmertest* library in R) results on effects of tide height on depth collected from fish tags, in which ID and date were treated as random factors. (DOCX)

S1 Video Seagrass at rocky bottom around Ping Island videoed by the LBV150-4. (MP4)

S2 Video Natural reef bottom around Ping Island videoed by the LBV150-4. (MP4)

S3 Video Boulder, cobble and gravel bottoms around Ping Island videoed by the LBV150-4. (MP4)

S4 Video Sandy bottom around Ping Island videoed by the LBV150-4. (MP4)

S5 Video Cement reefs around Ping Island videoed by the stereo-DOV. (MP4)

S6 Video A sunken steel vessel around Ping Island videoed by the stereo-DOV. (MP4)

S7 Video A sunken wooden vessel around Ping Island videoed by the stereo-DOV. (MP4)
