# Supplementary figures and images for: Short-Term Fidelity, Habitat Use and Vertical Movement Behavior of the Black Rockfish Sebastes schlegelii as Determined by Acoustic Telemetry
Source: PLoS One. 2015 Aug 31;10(8):e0134381. doi: 10.1371/journal.pone.0134381 (PMC4556453; doi:10.1371/journal.pone.0134381)

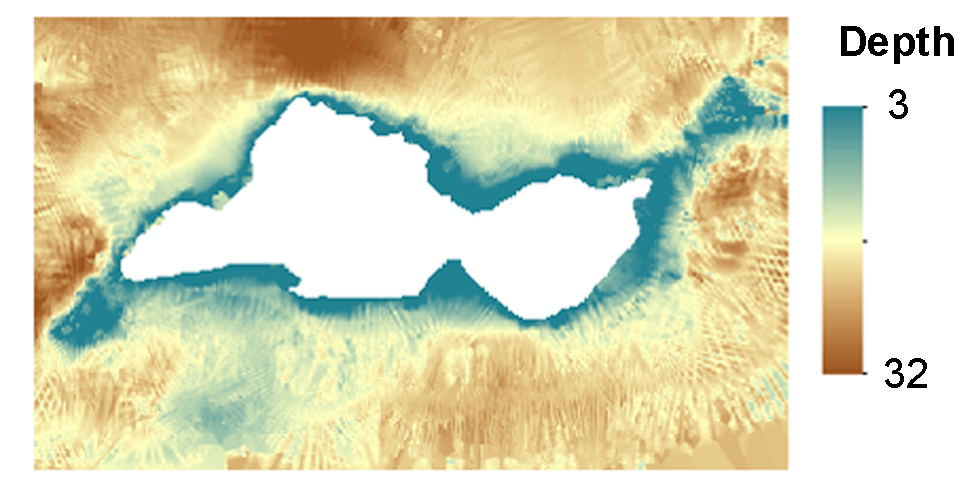

Supplement: S1 Fig — (TIF) [file pone.0134381.s001.tif]

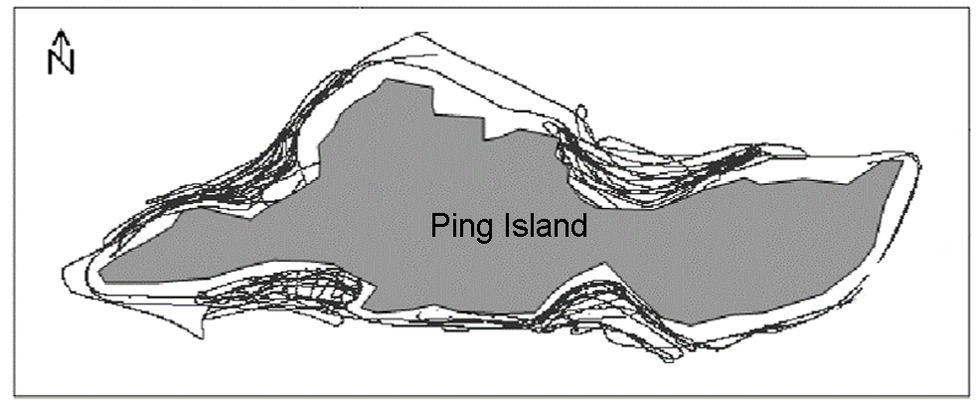

Supplement: S3 Fig — (TIF) [file pone.0134381.s003.tif]

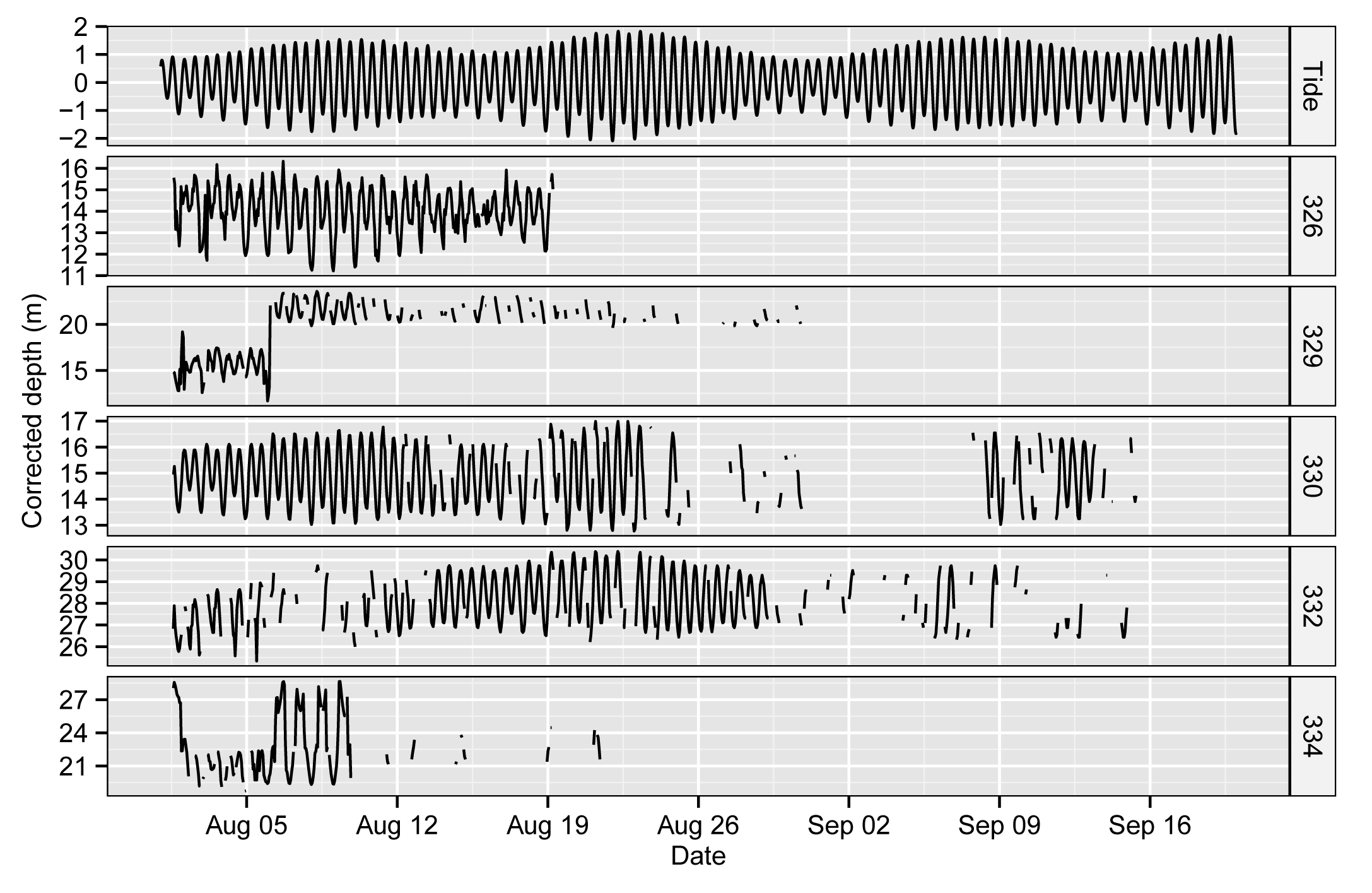

Supplement: S4 Fig — (TIF) [file pone.0134381.s004.tif]

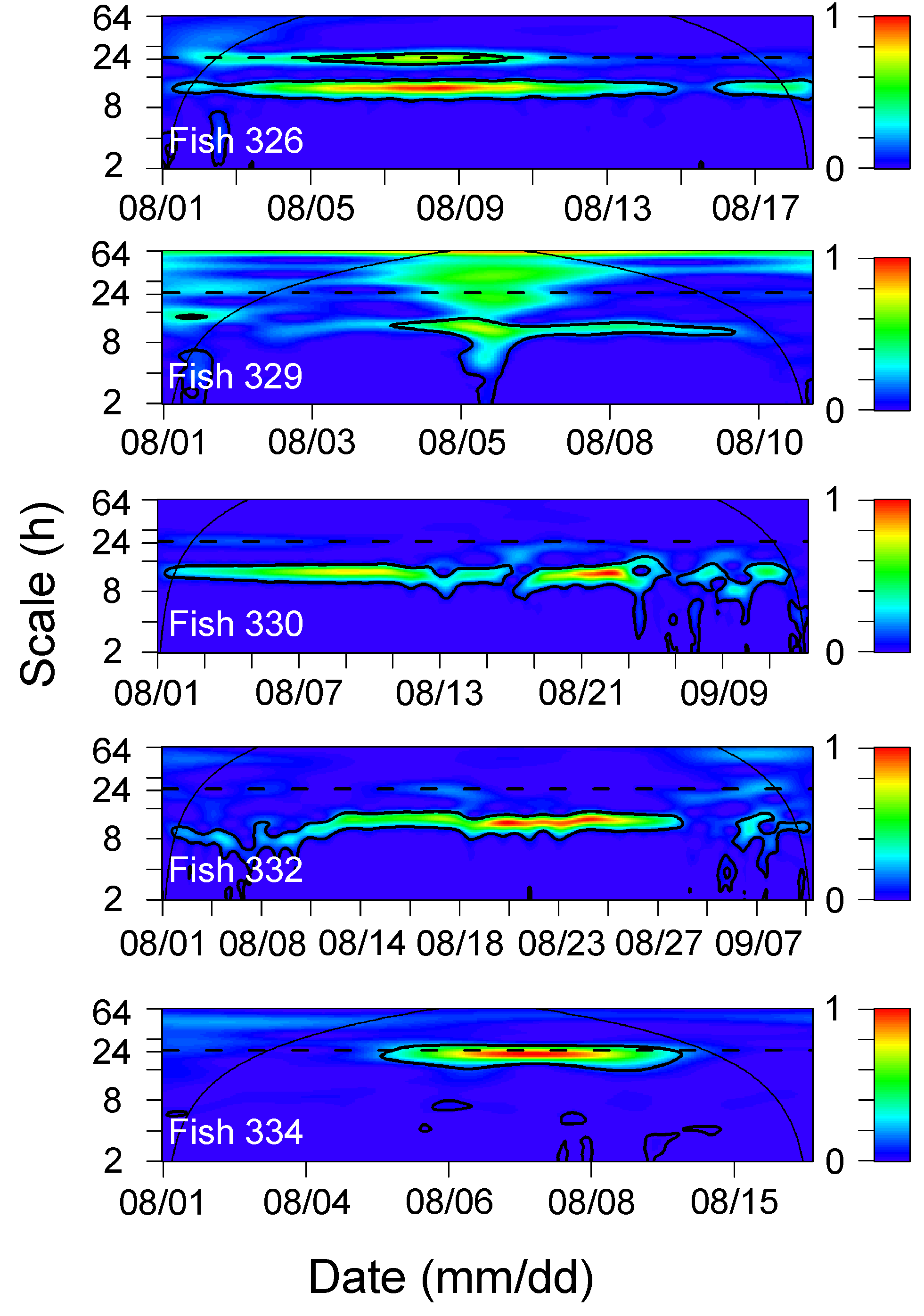

Supplement: S5 Fig — (TIF) [file pone.0134381.s005.tif]
