# Supplementary material for: Short-Term Fidelity, Habitat Use and Vertical Movement Behavior of the Black Rockfish Sebastes schlegelii as Determined by Acoustic Telemetry
Source: PLoS One. 2015 Aug 31;10(8):e0134381. doi: 10.1371/journal.pone.0134381 (PMC4556453; doi:10.1371/journal.pone.0134381)
Supplement: S1 Table — (DOCX) [file pone.0134381.s006.docx]

**S1 Table. The linear mixed model (in the *lmertest* library in R) results on effects of tide height on depth collected from fish tags, in which ID and date were treated as random factors.** Depth values collected from tagged fish were closely related to predicted tide height (*P* < 0.001).

|  | Estimate | Std. Error | t value | *P* (> \|t\|) |
| --- | --- | --- | --- | --- |
| (Intercept) | 0.21 | 1.71 | 12.10 | < 0.001 |
| Tide height | 1.01 | 0.01 | 101.50 | < 0.001 |
